# The role of decentering and self-compassion in self-esteem regulation: how meditation and metacognition shape the use of self-esteem regulation strategies

**DOI:** 10.3389/fpsyg.2026.1791258

**Published:** 2026-07-10

**Authors:** Baran Kahraman, Lena Rader, Siegfried Gauggel, Nina Doernberg, Verena Mainz, Lorenz Weise, Barbara Drueke

**Affiliations:** 1 Institute of Medical Psychology and Medical Sociology, University Hospital RWTH Aachen, Aachen, Germany; 2 Zentrum für Psychotherapie gGmbH, Chemnitz, Germany; 3 Institute for Translational Neuroscience and Clinical Psychology, University Hospital RWTH Aachen, Aachen, Germany

**Keywords:** metacognition, self-esteem, self-regulation, compassion, meditation

## Abstract

**Introduction:**

Previous research has demonstrated that individuals’ levels of self-esteem strongly influence how they regulate and protect their self-esteem. However, it remains unclear to what extent these regulatory strategies are shaped by trainable psychological processes such as decentering and self-compassion.

**Methods:**

Two studies were conducted to investigate the role of decentering and self-compassion in self-esteem regulation. Study 1 (*n* = 230) examined whether decentering and self-compassion mediate the relationship between trait self-esteem and self-esteem regulation strategies. In addition, differences between experienced meditators and non-meditators in self-esteem, decentering, self-compassion, self-affirmation and self-protection were explored. Study 2 (*n* = 66) employed a randomized controlled design to test the feasibility and preliminary short-term effects of two brief guided audio meditations intended to enhance decentering and self-compassion.

**Results:**

In Study 1, multiple regression analyses revealed that decentering was associated with higher self-affirmation, while self-compassion was linked to lower self-protection. Conditional process path analysis further showed that self-esteem was positively associated with both decentering and self-compassion and had a significant indirect effect on self-protection via self-compassion. Moreover, compared to non-meditators, meditators reported higher levels of self-esteem, decentering, and self-compassion. In Study 2, both brief audio-based interventions led to increases in self-compassion and decentering relative to the control condition, with the decentering intervention showing the strongest effects on post-intervention outcomes.

**Discussion:**

The findings indicate that decentering and self-compassion can be modifiable through brief meditative practices and may play an important role in the regulation of self-esteem. Enhancing these processes may represent a promising target for psychological interventions aimed at improving self-esteem regulation and resilience in individuals vulnerable to mental health problems.

## 1 Introduction

Self-esteem refers to the global assessment of one’s self based on perceived norms, competence and social evaluations (Brown and Zeigler-Hill, 2017; MacDonald, 2012; Cast and Burke, 2002). In its low expression, self-esteem is a well-documented risk factor for depressive and other mental disorders and is linked to internalizing and externalizing behaviors as well as social withdrawal (Orth et al., 2009; Keane and Loades, 2017; Donnellan et al., 2005). Given its central role in psychological well-being, self-esteem is increasingly regarded as a modifiable factor that can be targeted through psychotherapeutic and contemplative interventions (see Zeigler-Hill, 2011; Bhattacharya et al., 2023).

To protect their self-esteem, individuals use a wide array of different self-esteem strategies (Leary, 2007; Hepper et al., 2010). One key factor that is known to shape which strategies people rely on is their own level of self-esteem (Hepper et al., 2010; Hepper, 2016). People with high self-esteem typically engage in self-affirmation or self-enhancement strategies, such as recalling personal strengths or emphasizing valued aspects of the self, whereas those with low self-esteem are more likely to rely on self-protection strategies (Hepper et al., 2010).

Self-protection strategies, which are defensive cognitive modifications and behavioral responses aimed at reducing threats to one’s own self-worth, include, among others, defensiveness, external attribution, downward social comparison, and self-handicapping (see Hepper et al., 2010; Harris et al., 2019; Lannin et al., 2021; Rader et al., 2025). While they provide short-term relief from self-esteem relevant threats and enable swift coping in acute aversive threat contexts (Cohen and Sherman, 2014), they are associated with defensive pessimism (Alicke and Sedikides, 2009) and reduced openness to growth-relevant information, potentially constraining long-term self-development (Sherman and Hartson, 2011; Cohen and Sherman, 2014).

By contrast, self-affirmation strategies are characterized by a less reactive and threat-focused approach and instead promote adaptation through the affirmation of alternative self-domains (Steele, 1988). They are considered adaptive because they ultimately foster a broader sense of global self-integrity, support psychological flexibility, and promote long-term resilience (Steele, 1988; Cohen and Sherman, 2014). In the long-term, cultivation in self-affirmation strategies leads to an increased awareness that the self-concept consists of multiple facets and is not exclusively centered on the threatened self-domain (Steele, 1988; Cohen and Sherman, 2014; Critcher and Dunning, 2015).

Despite the importance of these regulatory mechanisms as part of the broader psychological immune system (Sedikides, 2021; Howell, 2017), relatively little is known about additional psychological factors that determine which of these strategies individuals employ.

Evidence suggests that decentering, a metacognitive process to observe one’s thoughts and emotions as transient mind-made mental events, may play a critical role in the usage of these strategies (Bernstein et al., 2015; Naragon-Gainey et al., 2020; Rader et al., 2025). Decentering enhances individuals’ capacity to distance themselves from unpleasant emotions and thoughts, ultimately fostering a more detached and balanced self-view (Shepherd et al., 2016; De Oliveira et al., 2024; Lebois et al., 2015). Recent research shows that self-esteem may predict this important metacognitive process (i.e., decentering) which in turn is associated with self-affirmations (Rader et al., 2025). In this context, higher decentering is linked to a better internalization of self-resources, functional thought patterns, psychological flexibility and adaptive emotion regulation processes (Bennett et al., 2021; Bernstein et al., 2015) indicating that decentered people may have easier accessibility or a more natural tendency to use adaptive self-esteem strategies (Rader et al., 2025). By contrast, low levels of decentering are associated with psychological disorders (e.g., depression) and distorted self-views (Bennett et al., 2021; Kessel et al., 2016; Drueke et al., 2023; Tapper and Turner, 2018), demonstrating and underlining that the cultivation of the decentering process is integral to foster adaptive and functional self-regulatory processes (Rader et al., 2025; Shepherd et al., 2016; Shepherd, 2014).

Another key construct relevant to self-esteem regulation that may influence individuals’ choice in self-esteem strategies represents self-compassion. Rooted in Buddhist tradition, self-compassion is a trainable attitude (Strauss et al., 2016; Quaglia et al., 2021; Fraser et al., 2023) characterized by self-kindness, recognition of common humanity, and mindful awareness of one’s suffering (see Neff, 2023).

Self-compassion promotes resilience and confers many of the psychological benefits traditionally associated with high self-esteem, while avoiding the well-documented drawbacks, such as reliance on social comparison and narcissism (Neff, 2023; Neff, 2009; Marshall et al., 2015). High self-compassion has been associated with lower psychopathology, lower self-protection tendencies, greater well-being, and improved emotion regulation (Neff, 2011; Neff, 2023; Poli et al., 2022; Harwood and Kocovski, 2017). Moreover, self-compassion has been described as a valid and robust predictor for well-being, life satisfaction and the usage of adaptive emotion regulation strategies (Muris and Otgaar, 2023; Kamalinasab and Mohammadkhani, 2018; Fraser et al., 2023), making it an especially important target for psychological and preventive interventions.

Although both decentering and self-compassion are conceptually related, they represent distinct psychological constructs. From a theoretical perspective, decentering is understood as a fundamental metacognitive mechanism and process that can function as a precursor to certain self-regulatory responses. By enabling individuals to disengage from immediate identification with self-threatening thoughts and emotions, decentering creates psychological space for adaptive self-regulation and subsequent downstream processes. Self-compassion is one such downstream response. Accordingly, higher levels of decentering may facilitate more frequent self-compassionate responding by reducing identification with self-critical cognitions and associated affective distress (see Bernstein et al., 2015; Neff, 2023).

Research shows that both constructs, decentering and self-compassion, can be cultivated through meditation, which has consistently been shown to enhance self-regulation and self-esteem (Wisner et al., 2010; Pepping et al., 2013; Morley and Fulton, 2020). Traditional meditation programs such as Mindfulness-Based Stress Reduction (MBSR; Kabat-Zinn et al., 1992) and Loving-Kindness Meditation (LKM; Fredrickson et al., 2008) effectively promote these processes, though most interventions are long-term. While previous research has examined decentering and self-compassion as mediators in the context of mindfulness-based interventions (e.g., Maloney, 2023; Roca et al., 2021), less is known about how these processes compare to each other as brief single-session mediations, particularly in relation to active control conditions.

Building on prior findings, recent evidence indicates that brief meditation sessions, delivered digitally or in-person, can improve psychological well-being and self-regulatory capacities (Flett et al., 2019; Mandlik et al., 2024). Against this background, the present research pursued two complementary objectives.

Study 1 examined how decentering and self-compassion relate to self-esteem and self-esteem regulation strategies. Although mindfulness-based cognitive therapy (MBCT) and compassion-focused therapy (CFT) target self-referential processing, self-esteem has received comparatively little attention as a mechanistic outcome (see Britton et al., 2021). A better understanding of how multiple self-related processes interact, may help to clarify clinically relevant mechanisms of change. Accordingly, the present study, which was correlational in nature, investigated whether decentering and self-compassion mediate the relationship between trait self-esteem and individuals’ preferred self-esteem regulation strategies. Based on prior evidence, we hypothesized that higher self-esteem would be associated with greater decentering and self-compassion, and that both processes would, in turn, be linked to more adaptive self-esteem regulation strategies, such as self-affirmation rather than self-protection.

Study 2 extended this work by experimentally testing the short-term malleability of these same processes using brief guided meditation. Two audio-based interventions were compared: a mindfulness-focused meditation targeting decentering and a Loving-Kindness Meditation (LKM) targeting self-compassion. These were selected because they map onto the two mechanisms identified in Study 1 and represent established but theoretically distinct pathways within MBCT and CFT frameworks. Based on prior theoretical assumptions that decentering and self-compassion are highly correlated, we expected that both interventions would lead to immediate increases in state levels of both constructs following a single session. Furthermore, we expected that individual differences in trait self-esteem, decentering, and self-compassion would influence these effects.

This dual approach allowed for both correlational and experimental insights into how trainable psychological processes, decentering and self-compassion, shape self-esteem regulation. It also addressed the open question of whether these constructs, although closely related, can be empirically differentiated within the context of contemplative practice.

## 2 Study 1

Study 1 was associational and exploratory in nature and designed to assess how decentering and self-compassion relate to individuals’ self-esteem and their subsequent use of regulation strategies. Further, we were interested in potential differences between individuals with and without meditation experience. Therefore, we hypothesized that (1) decentering and self-compassion mediate the relationship between self-esteem and self-esteem regulation strategies. Specifically, higher self-esteem was expected to associate with greater decentering and self-compassion, which in turn would be associated with increased use of adaptive self-affirmation strategies and decreased reliance on defensive self-protection. Additionally, (2) individuals with prior meditation experience were expected to report higher levels of self-esteem, decentering and self-compassion and, consequently, to show a greater tendency to engage in adaptive self-regulation strategies. Meditation experience was furthermore expected to moderate the relationship between self-esteem and self-esteem strategies.

### 2.1 Methods

#### 2.1.1 Participants and ethical considerations

Two hundred thirty healthy German-speaking participants (*n* = 125 meditators, *n* = 105 non-meditators) aged between 18 and 65 years (*M* = 40.98 ± 15.48) were recruited using online advertisements and flyers distributed at local communities, the university and two cooperating meditation centers. Meditators of the sample (*Mage* = 49.84 ± 12.36) were on average older than non-meditators (*Mage* = 30.43 ± 11.78), see Table 1). 27.4% (*n* = 63) of the sample consisted of individuals between the ages of 18 to 25 years old, while 69.1% (*n* = 159) were between the ages of 26 and 65. 3.5% (*n* = 8) of the sample were 66 years or older. 76.1% of participants in the total sample identified as female while 23% identified as male and 0.9% as diverse. 33.9% of the sample reported to have a university degree (see Table 1).

**Table 1:** Sample characteristics for the total sample and both subgroups (meditators vs. non-meditators).

Variable	Total sample (*n* = 230)	Meditators (*n* = 125)	Non-meditators (*n* = 105)
Age (in years)	40.98 ± 15.48	49.84 ± 12.36	30.43 ± 11.78
Gender			
Female	175 (76.1%)	95 (76%)	80 (76.2%)
Male	53 (23%)	29 (23.3%)	24 (22.9%)
Diverse	2 (0.9%)	1 (0.8%)	1 (1%)
Educational level			
High School	17 (7.4%)	11 (8.8%)	38 (36.2%)
Univ. Qualification	65 (28.3%)	33 (26.4%)	19 (18.1%)
Vocational	40 (17.4%)	21 (16.8%)	35 (33.3%)
Bachelor	49 (21.3%)	14 (11.2%)	8 (7.6%)
Master	24 (10.4%)	16 (12.8%)	1 (1.0%)
PhD	5 (2.2%)	4 (3.2%)	4 (3.8%)
Other	30 (13.0%)	26 (20.8%)	—

Age (Mean ± sd); gender, education level (frequency and percentage).

Within the subsample of meditators (*n* = 125), 66.4% reported having previously participated in mindfulness-based meditation several years prior to the present study, whereas 16.0% reported prior engagement in yoga-based or body-focused meditation practices. Deconstructive types and constructive meditation types (see Schlosser et al., 2019) were adhered to by only 5.6% and 4.0% of the sample, respectively. Eight percent of participants classified their meditation practice as “other.” The average frequency of meditation session habitually followed per week was *M* = 4.70 ± 2.99. The average duration of such a meditation session was *M* = 21.44 ± 11.67 min, showing that the meditation subsample consisted of experienced meditators.

Exclusion criteria consisted of a current or past diagnosis of mental or neurological disorders and poor language proficiency based on self-report. All hypotheses and the corresponding statistical analyses were preregistered on PsychArchives. As indicated in the preregistration, a variable in addition to decentering and self-compassion, namely other-compassion, measured via the compassion for others scale (COS-7, Schlosser et al., 2023) was originally planned for inclusion. However, other-compassion (COS) was finally excluded from the main model due to considerations of model complexity and to simplify the presentation. Comparing models with and without the COS showed that this exclusion did not affect the main results or the mediation effects. Accordingly, only decentering and self-compassion are reported in the present article, while the model including other-compassion as a mediator is fully documented in the Supplementary Figure S1 for transparency. The Supplement also briefly notes that other-compassion may relate differently to the strategies compared to self-compassion. The study was approved by the ethical board of the University Hospital of RWTH Aachen University (EK 24-120).

#### 2.1.2 Data collection

Participants were assessed online via the platform Qualtrics.^1^ In the introductory section of the survey, we provided information about the study, i.e., aim, methods, and permission of the participants to withdraw at any point. All participants gave their informed consent. First, participants were asked to provide information concerning demographic variables such as gender and age, but also information regarding their current psychological wellbeing and their meditation experience. Then, participants were asked to complete different questionnaires measuring their levels of self-esteem, decentering, and self-compassion. Finally, participants were asked about their self-esteem strategies (see “Materials and Measures”).

#### 2.1.3 Materials and measures

Self-esteem was measured using the German version of the Rosenberg Self-Esteem Scale (Rosenberg, 1965; Von Collani and Herzberg, 2003) consisting of 10 items on a 4-point Likert scale (1 = “strongly agree” to 4 = “strongly disagree”). A maximum score of 40 indicates very high global self-esteem. The Self-esteem scale is a valid and widely used instrument to quantify individual global self-worth and shows an internal consistency of *α* = 0.84 (from Von Collani and Herzberg, 2003).

Decentering was assessed using the German version of the Experiences Questionnaire (Fresco et al., 2007; Gecht et al., 2014). Only the 13 items loading on the Decentering factor were used to prevent confounding with the rumination factor and to isolate the construct of decentering in its distinct form. A maximum score of 65 indicates very high decentering. The Items are answered on a 5-point Likert scale (1 = “never,” 5 = “all the time”). The internal consistency shows *α* = 0.83 and *ω* = 0.88 (Rader et al., 2023; Fresco et al., 2007).

Self-Compassion was measured through the German short form of the Self-Compassion Scale (SCS, Hupfeld and Ruffieux, 2011). The short form consists of 12 items on a 5-point Likert scale (1 = “almost never” to 5 = “almost always”) and measures in which way people generally show self-compassion to themselves. A maximum score of 60 indicates very high self-compassion. The internal consistency of the short version is *α* = 0.84 (Hupfeld and Ruffieux, 2011).

Other-Compassion was measured using the Other-Compassion Scale (COS-7) by Schlosser et al. (2023) consisting of 7-items on a 7-point Likert scale (1 = “not at all true of me,” 7 = “very true of me”). A maximum score of 49 indicates very high other-compassion. It is a relatively new but valid scale measuring people’s inclination for other-compassion. The internal consistency of the scale is *α* = 0.89 (Schlosser et al., 2023).

To measure Self-affirmation, we used all 13 items of the Spontaneous Self-affirmation Measure (SSAM) consisting of the strengths, values and social relations subscales (Harris et al., 2019). The Spontaneous Self-affirmation Measure operationalizes in which way people spontaneously participate in self-affirmations. The items are on a 7-point Likert scale (1 = “strongly disagree, 7 = “strongly agree).” A maximum score of 91 indicates very high self-affirmation tendencies. The internal consistency shows *α* = 0.93 (Harris et al., 2019).

Self-protection was measured using the Defensiveness subscale of the Self-Enhancement and Self-Protection Scale developed by Hepper et al. (2010). This validated subscale consists of 18 items that capture a range of commonly employed self-protection strategies. Participants respond to each item on a 6-point Likert scale ranging from 1 (“not at all characteristic of me”) to 6 (“very characteristic of me”). A maximum score of 108 indicates very high self-protection tendencies. According to Hepper et al. (2010), the internal consistency of the subscale is high, with Cronbach’s alpha values reported as *α* = 0.83 and *α* = 0.86 across different studies (see Hepper et al., 2010).

Meditation experience was assessed based on items adapted from Schlosser et al. (2019) (see Appendix S1 Q6–Q9 in Schlosser et al., 2019). At the beginning of the questionnaire, participants were asked about their general meditation habits, specifically whether they regularly meditated at least once a week. Those who answered “No” were classified as non-meditators and directed to continue with the subsequent questionnaires. Participants who responded “Yes” were asked to provide more detailed information about their meditation practice. In addition, participants were asked whether they engaged in body-focused techniques such as yoga or other types of meditation. Altogether, meditation experience was operationalized using five core questions.

### 2.2 Data analysis

#### 2.2.1 Data integrity checks

Data analysis was conducted using the statistical software R^2^. During the Qualtrics-based assessment, two data integrity (attention) checks were embedded within the questionnaires. Specifically, attention-check items were placed midway through the Defensiveness subscale and the Experiences Questionnaire. Participants were instructed to select “not at all characteristic of me” for Item 18 of the Defensiveness subscale and “never” for Item 13 of the Experiences Questionnaire. Participants who failed to select the correct response on both attention-check items were excluded from further analyses. In addition, the data were manually screened for outliers and straightlining (i.e., participants exhibiting zero variance across all questionnaire items). No participant had to be excluded based on these criteria (i.e., outliers and straightliners) and the analysis was conducted based on 230 participants.

#### 2.2.2 Multiple regression analyses

To examine the direct associations between decentering, self-compassion, and self-esteem with self-affirmation and self-protection, we conducted multiple regression analyses. We examined each outcome variable (self-affirmation and self-protection) by entering all three predictors (self-esteem, decentering, and self-compassion) simultaneously as sum scores. This allowed us to estimate the unique contribution of each predictor while controlling for the other two. These analyses provided a baseline understanding of direct effects and allowed comparison with the subsequent mediation model to assess indirect and total effects.

#### 2.2.3 Conditional process (path) analysis

Mediation was evaluated following Zhao et al.’s (2010) framework, which defines mediation as the presence of a significant indirect effect, using Hayes (2017) regression-based conditional process approach with bootstrapped (bootstrap = 5,000) confidence intervals.

**Figure 1:**
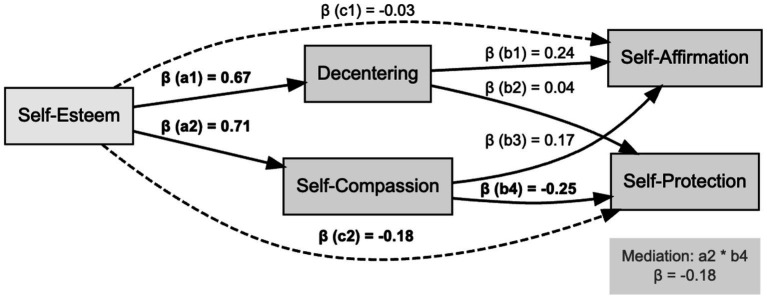
Mediation model of study 1 with corresponding path coefficients. The figure shows the path analysis and the mediation of self-compassion in the relationship between self-esteem and self-protection (a2*b4, *β* = −0.18). Significant paths are indicated by bold lines.

In the specified model, self-esteem served as the independent variable, decentering and self-compassion were included as parallel mediators, and self-affirmation and self-protection were the outcome variables (see Figure 1).

Indirect effects were estimated as the product of a- and b-paths and considered statistically significant if the 95% confidence interval did not include zero. Effects were interpreted based on the presence and direction of significant direct and indirect paths, without distinguishing between full and partial mediation. All analyses were conducted and interpreted using the analytic approach following PROCESS conventions, which implements ordinary least squares regression rather than covariance-based structural equation modeling. Therefore, global fit indices were not used. The sample size exceeded recommended thresholds for detecting medium-sized indirect effects (Boomsma, 1982, 1985, as cited in Wolf et al., 2013; Russell et al., 1998).

In addition, variance inflation factors (VIFs) were computed to assess multicollinearity between decentering and self-compassion, and their correlation was examined to evaluate potential construct overlap. Moreover, to test the robustness of the mediation effects for the full path model, separate simple mediation models were estimated for each mediator (i.e., decentering and self-compassion).

#### 2.2.4 Analyses of covariance and moderated mediation

To examine whether levels of meditation experience were associated with differences in study variables, we conducted a one-way multivariate analysis of covariance (MANCOVA) to test for overall group differences across all dependent variables simultaneously. Age was included as a continuous covariate, and gender was included as a categorical control factor. When the MANCOVA yielded a significant multivariate effect, follow-up univariate analyses of covariance (ANCOVAs) were conducted for each dependent variable to identify specific group differences. Meditation experience (yes vs. no) served as the independent variable, and decentering, self-compassion, self-esteem, self-affirmation, and self-protection were included as dependent variables. Pillai’s Trace was selected as the test statistic for its robustness to violations of homogeneity and unequal sample sizes.

Finally, we conducted a series of moderated mediation analyses to examine whether indirect effects of self-esteem on self-esteem regulation strategies (i.e., self-affirmation and self-protection) via decentering and self-compassion were systematically moderated by meditation experience. The analysis was performed using Hayes’ PROCESS macro (Version 3.0; Hayes, 2017), specifying Model 7. All indirect effects were estimated using 5,000 bootstrap samples (95% confidence intervals). In these analyses, meditation experience was specified as a dichotomous moderator (0 = no prior meditation experience, 1 = prior meditation experience) of the path from self-esteem (independent variable) to each mediator, corresponding to moderation of the a-path. To capture potential differences across mediators and outcomes, four separate moderated mediation analyses were conducted using PROCESS Model 7: (1) Decentering as mediator predicting self-affirmation, (2) Decentering as mediator predicting self-protection, (3) Self-Compassion as mediator predicting self-affirmation, and (4) Self-Compassion as mediator predicting self-protection. This approach allowed us to estimate conditional indirect effects of self-esteem on each outcome through each mediator at different levels of meditation experience, as well as the index of moderated mediation.

### 2.3 Results

#### 2.3.1 Multiple regression analyses

Decentering was significantly linked to higher scores in self-affirmation (*ß* = 0.56, 95% CI [0.08, 1.04], *p* < 0.05), whereas self-compassion (*ß* = 0.32, 95% CI [−0.10, 0.73], *p* = 0.13) and self-esteem (*ß* = −0.10, 95% CI [−0.64, 0.45], *p* = 0.72) were not significant predictors in the regression model. The model accounted for 12.6% of the variance in self-affirmation (R^2^ = 0.126, *F* (3, 225) = 10.85, *p* < 0.001).

For self-protection, self-compassion (*ß* = −0.35, 95% CI [−0.64, −0.06], *p* < 0.05) and self-esteem (*ß* = −0.40, 95% CI [−0.79, −0.02], *p* < 0.05) associated significantly with lower scores, whereas decentering was not significant (*ß* = 0.07, 95% CI [−0.27, 0.41], *p* = 0.69). This model explained 14.4% of the variance in self-protection [R^2^ = 0.144, *F* (3, 225) = 12.6, *p* < 0.001].

#### 2.3.2 Conditional process (path) analysis

Path analysis revealed that self-esteem was significantly related to both decentering (*β* = 0.67, 95% CI [0.60, 0.73]) and self-compassion (*β* = 0.71, 95% CI [0.65, 0.77]), showing that higher self-esteem was associated with higher levels of both mediators. Self-compassion was further associated with lower self-protection strategies (*β* = −0.25, 95% CI [−0.45, −0.04]) All other direct associations, including those between self-compassion and self-affirmation (*β* = 0.17, 95% CI [−0.09, 0.42]), decentering and self-affirmation (*β* = 0.24, 95% CI [−0.05, 0.49]) and decentering and self-protection (*β* = 0.04, 95% CI [−0.21, 0.26]), were non-significant.

Following the results of the a and b-paths, mediation analyses revealed a significant indirect effect of self-compassion on self-protection (*β* = −0.18, 95% CI [−0.32, −0.03]), whereas its indirect effect on self-affirmation was not significant (*β* = 0.12, 95% CI [−0.07, 0.30]). Moreover, no significant indirect effects emerged for decentering, either on self-affirmation (*β* = 0.16, 95% CI [−0.03, 0.32]) or on self-protection (*β* = 0.03, 95% CI [−0.14, 0.17]). See Figure 1 for detailed results.

Regarding multicollinearity, decentering and self-compassion were strongly correlated (*r* = 0.78), although variance inflation factors (VIF = 2.55) indicated acceptable levels of multicollinearity.

Robustness checks via simplified mediator models showed a largely consistent pattern of effects for the full model, with the exception that single-mediator models yielded significant associations and indirect effects involving self-affirmation that were not significant in the parallel multiple-mediator model.

#### 2.3.3 Analyses of covariance and moderated mediation

The multivariate analysis of covariance (MANCOVA) showed a significant multivariate effect for meditation experience, *Pillai’s Trace* = 0.127, *F* (5, 221) = 6.43, *p* < 0.001 as well as for age, *Pillai’s Trace* = 0.128, *F* (5, 221) = 6.50, *p* < 0.001, and gender, *Pillai’s Trace* = 0.100, *F* (10, 444) = 2.35, *p* = 0.011.

Follow-up univariate analyses of covariance (ANCOVAs) revealed significant effects of meditation experience on self-esteem, *F* (1, 225) = 11.22, *p* < 0.001, *η^2^p* = 0.048, decentering, *F* (1, 225) = 24.13, *p* < 0.001, *η^2^p* = 0.097, and self-compassion, *F* (1, 225) = 26.93, *p* < 0.001, *η^2^p* = 0.107. The effects on self-protection, *F* (1, 225) = 3.74, *p* = 0.054, *η^2^p* = 0.016, and self-affirmation, *F* (1, 225) = 0.02, *p* = 0.897, *η^2^p = 0*.00, were not significant.

**Figure 2:**
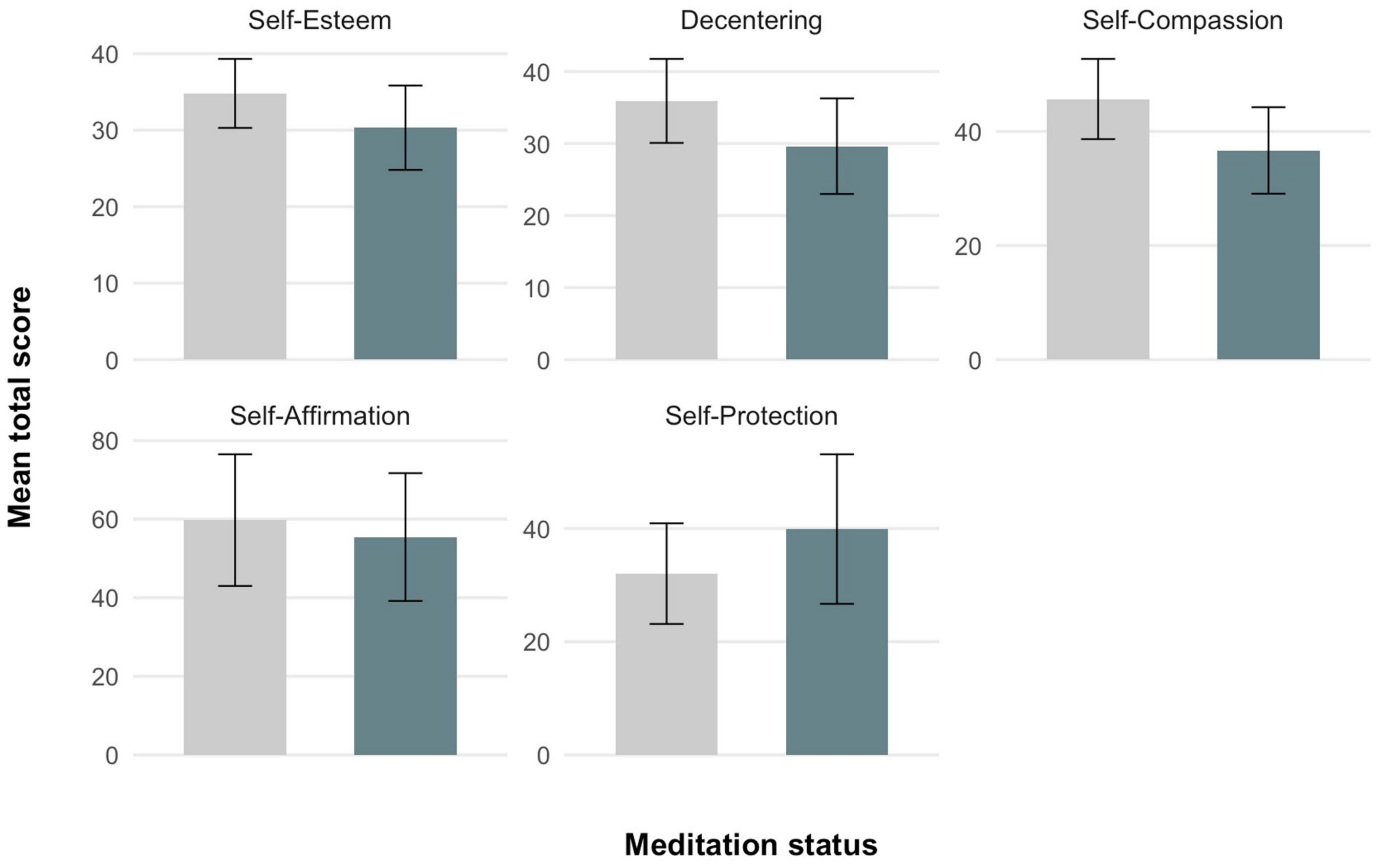
Observed means (unadjusted) and standard deviations for meditators (grey) and non-meditators (blue) in the measured variables.

Age significantly predicted all dependent variables in univariate regressions. Effects were positive for all constructs except self-protection, which showed a negative association. Although estimated marginal means indicated generally higher values for men compared to women across most dependent variables except for self-affirmation, pairwise comparisons were not significant for most outcomes besides decentering. Accordingly, the significant multivariate effect of gender was not consistently reflected at the univariate level (see Figure 2; Tables 2, 3).

**Table 2:** Means (unadjusted) and standard deviations for the total sample and subsamples (meditators and non-meditators).

Group	Total sample *N* = 230	Meditators *n* = 125	Non-Meditators *n* = 105
	M ± SD	M ± SD	M ± SD
Dependent variables			
Self-esteem	32.75 ± 5.46	34.80 ± 4.50	30.31 ± 5.51
Decentering	33.02 ± 6.95	35.89 ± 5.84	29.63 ± 6.63
Self-compassion	41.56 ± 8.54	45.68 ± 7.02	36.66 ± 7.57
Self-affirmation	57.76 ± 16.6	59.73 ± 16.7	55.42 ± 16.2
Self-protection	35.61 ± 11.7	32.02 ± 8.88	39.89 ± 13.2

**Table 3:** Results of the analyses of covariance with meditation experience as a factor controlled for age and gender.

Dependent variable	*F*-value	*p*	η^2^	Group difference
Self-esteem	11.22	<0.001	0.04	−4.48
Decentering	24.13	<0.001	0.09	−6.25
Self-compassion	26.93	<0.001	0.10	−9.01
Self-affirmation	0.02	=0.897	0.00	−4.30
Self-protection	3.74	=0.054	0.01	7.87

Regarding the moderated mediation analyses, no significant interactions between self-esteem and meditation experience emerged across models, and none of the indices of moderated mediation reached significance, as all 95% bootstrap confidence intervals included zero, indicating no systematic moderation of the indirect effects.

However, several conditional indirect effects were significant. Decentering mediated the association between self-esteem and self-affirmation both among participants with meditation experience (ab = 0.64, 95% CI [0.25, 1.02]) and those without meditation experience (ab = 0.52, 95% CI [0.20, 0.84]), whereas no significant indirect effects via decentering were observed for self-protection. In addition, self-compassion mediated the association between self-esteem and self-protection, with comparable conditional indirect effects for participants with (ab = −0.32, 95% CI [−0.57, −0.11]) and without meditation experience (ab = −0.28, 95% CI [−0.49, −0.10]). Furthermore, self-compassion likewise mediated the association between self-esteem and self-affirmation in both meditators (ab = 0.56, 95% CI [0.18, 0.97]) and non-meditators: (ab = 0.48, 95% CI [0.15, 0.84]).

### 2.4 Discussion study 1

The present study investigated how decentering and self-compassion, influence self-esteem regulation and whether these processes differ as a function of prior meditation experience. Across multiple analytic approaches, a coherent pattern emerged, highlighting distinct yet complementary roles of decentering and self-compassion in shaping and influencing adaptive and defensive regulatory strategies.

At the level of baseline associations (multiple regression analyses), decentering was significantly linked to self-affirmation (*β* = 0.56), suggesting that the capacity to adopt a distanced and non-identifying perspective on internal experiences may be associated with more adaptive, affirming responses to self-relevant information. In contrast, self-compassion was particularly relevant for the reduction of self-protective strategies (*β* = −0.35), indicating that a kind and accepting stance toward oneself may be associated with fewer defensive reactions when self-esteem is threatened. These findings support the notion that self-esteem regulation is not a unitary process but rather relies on qualitatively different (meta)-cognitive mechanisms depending on the regulatory goal.

The path analytic model further clarified these relationships by positioning self-esteem as an important antecedent of both decentering and self-compassion. Higher levels of self-esteem were associated with greater access to both capacities, underscoring the idea that self-esteem may function as a psychological resource that may enable more flexible and adaptive self-regulation. Within this model, self-compassion emerged as a significant mediator of the association between self-esteem and reduced self-protection, suggesting its role in attenuating defensive responses. In contrast, the expected indirect effects of decentering and self-compassion on self-affirmation were non-significant, although their consistently positive estimates were in line with theoretical expectations.

The moderated mediation analyses offered additional nuances and helped to provide additional insight into the observed path-analytical findings. Although meditation experience did not significantly moderate any of the modeled pathways, inspection of the conditional indirect effects revealed a more differentiated picture. Decentering significantly mediated the association between self-esteem and self-affirmation in both groups (meditators and non-meditators), despite its indirect effect falling just short of significance in the aggregated path model. A similar pattern emerged for self-compassion, which mediated not only the relationship between self-esteem and reduced self-protection as in the path analysis, but also the expected association between self-esteem and increased self-affirmation across both meditators and non-meditators (see Limitations section for further discussion).

Finally, group comparisons provided further additional context for our findings. Analyses of covariance controlling for age and gender indicated that meditators showed higher levels of self-esteem, decentering, and self-compassion. However, effects for self-affirmation and self-protection were attenuated after covariate adjustment, suggesting that these variables were at least in part influenced by demographic factors. Further interpretation of these patterns is provided in the General Discussion.

## 3 Study 2

Study 2 was a randomized controlled trial and assessed the feasibility and short-term psychological effects of two brief audio-guided meditation interventions in decentering and self-compassion. The preliminary study aimed to investigate whether changes in decentering and self-compassion can be measured after even a single audio session of either meditation practice. To the author’s knowledge, no quantitative study has systemically investigated the differential effects of decentering and self-compassion audio interventions in combination with an active control group. This study therefore served both as a preliminary test of the interventions’ efficacy and as an inquiry into their practical integration into everyday contexts.

Considering this, we hypothesized that (1) the brief decentering exercise would lead to immediate increases in state decentering and (2) the brief self-compassion exercise to immediate increases in state self-compassion. We also expected the interventions to produce overall higher decentering and self-compassion scores compared to the control group (3). Finally, we expected that pre-existing levels of self-esteem, decentering, and self-compassion would influence state outcomes and the effects of the interventions (4).

### 3.1 Methods

#### 3.1.1 Participants

**Figure 3:**
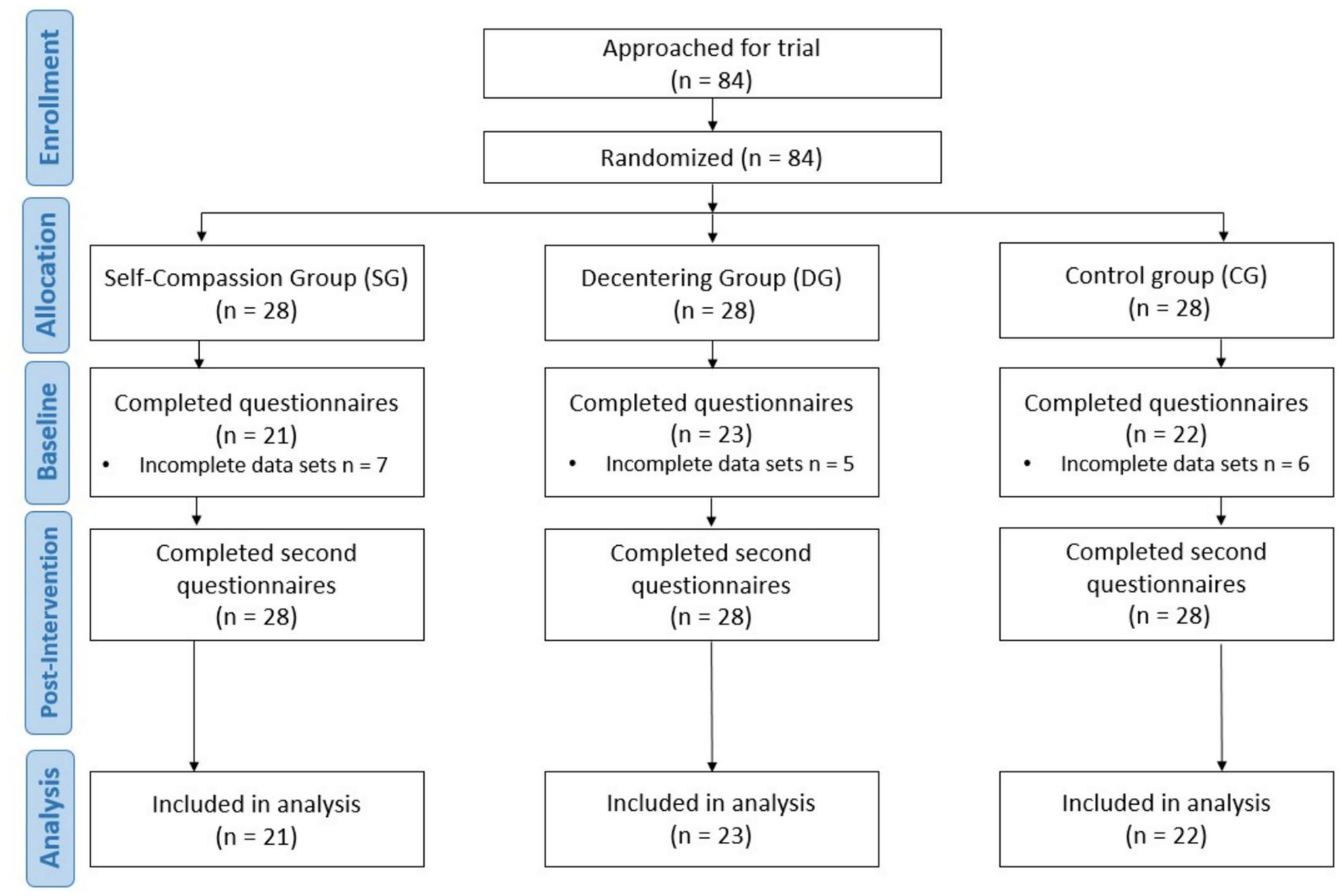
Consort flow chart (DG, decentering group; SG, self-compassion group, CG, control group).

Sixty six healthy German-speaking participants aged between 18 and 50 (*Mage* = 26.8 ± 6.89) years were recruited using online advertisements and flyers distributed at local communities and the university campus. Exclusion criteria consisted of a current or past diagnosis of mental or neurological disorders or an ongoing psychotherapy or medication for mental disorders based on self-report (see Figure 3 for an overview of the inclusion and exclusion of participants based on the CONSORT guidelines and Table 4 for a detailed overview for the age, gender, education and meditation distribution). All participants provided written informed consent and received financial compensation for their participation. The study was approved by the ethical board of the University Hospital of RWTH Aachen University (EK 24-418). The study was preregistered on PsychArchives.

**Table 4:** Sample characteristics for the total sample and each subgroup (Decentering: DG; self-compassion: SG; and control group: CG).

Variable	Total (*N* = 66)	DG (*N* = 23)	SG (*N* = 21)	CG (*N* = 22)
Age (years)	26.8 (6.89)	24.43 (4.76)	28.85 (7.92)	27.31 (7.26)
Gender				
Female	38 (57.6%)	13 (56.5%)	9 (42.9%)	16 (72.7%)
Male	28 (42.4%)	10 (43.5%)	12 (57.1%)	6 (27.3%)
Nationality				
German	56 (84.8%)	19 (82.6%)	21 (100%)	16 (72.7%)
Other	10 (15.2%)	4 (17.4%)	0 (0.00%)	6 (27.3%)
Meditation				
None	57 (86.4%)	20 (87%)	19 (90.5%)	18 (81.8%)
At least 1x week	9 (13.6%)	3 (13%)	2 (9.5%)	4 (18.2%)
Education				
High school	2 (3%)	1 (4.3%)	1 (4.8%)	0 (0.00%)
Univ. Qual.	29 (42.9%)	12 (52.2%)	6 (28.6%)	11 (50%)
Vocational	9 (13.6%)	3 (13%)	4 (19%)	2 (9.1%)
Bachelor	17 (26.8%)	5 (21.7%)	6 (28.6%)	6 (27.3%)
Master	5 (7.6%)	1 (4.3%)	3 (14.3%)	1 (4.5%)
PhD	1 (1.5%)	0 (0.00%)	0 (0.00%)	1 (4.5%)
Other	3 (4.5%)	1 (4.3%)	1 (4.8%)	1 (4.5%)

Age in years (Mean ± sd); Gender, nationality, meditation experience and education level (in frequency and percentage).

#### 3.1.2 Procedure

On the day of the examination, participants arrived at the laboratory and received standardized information about the experimental procedure. After providing informed consent, they completed a demographic questionnaire (e.g., age, gender). Subsequently, baseline assessments were conducted, including measures of trait decentering, trait self-compassion, and trait self-esteem, as well as state decentering and state self-compassion (see Section 3.1.3, Materials).

Then, participants were randomly allocated in a 1:1:1 ratio to one of three conditions (decentering, self-compassion, or control) using computer-generated block randomization with randomly alternating block sizes of 3, 6, and 9. The random allocation sequence was generated prior to data collection, and group assignments were concealed until the moment of allocation to minimize selection bias. The audio files were each approximately 15 min long. The participants were blinded to the conditions until the moment they heard the audio file. During the completion of the questionnaires and listening to the audio file, the examiner was not in the same room as the participants to minimize a possible examiner effect. After listening to the audio recordings, participants completed the post-intervention questionnaires assessing state decentering and state self-compassion. Finally, participants received a debriefing stating the intent of the study (see Figure 3 below for a visualization of the study procedure and Supplementary Table S1 for detailed information regarding the interventions). Across all groups, a total of 18 datasets were incomplete due to technical difficulties (self-compassion group: *n* = 7; decentering group: *n* = 5; control group: *n* = 6) and were therefore not included in the statistical analyses (see Figure 3).

#### 3.1.3 Materials

Trait self-esteem was measured using the German version of the Rosenberg Self-Esteem Scale (*α* = 0.84) consisting of 10 items (von Collani and Herzberg, 2003) on a 4-point Likert scale (1 = “strongly agree,” 4 = “strongly disagree”) while trait-decentering was measured using the German version of the Experience Questionnaire (*α* = 0.83, Fresco et al., 2007) consisting of 21 Items on a 5-point Likert scale (1 = “never,” 5 = “all the time”). For self-compassion we included the German short version of the Self-Compassion Scale consisting of 12 items on a 5-point Likert scale (1 = “almost never,” 5 = “almost always”) with a Cronbach’s alpha of *α* = 0.84 (SCS, Hupfeld and Ruffieux, 2011).

For the measurement of state-decentering, we used the Mind subscale of the State Mindfulness Scale (SMS-M) consisting of 15 items on a 5-point Likert scale (1 = “not at all,” 5 = “very much”) (Tanay and Bernstein, 2013, *α* = 0.95 for the Mind subscale). State self-compassion (SSCS-S) was measured through the short form of the state self-compassion scale (Neff et al., 2021, Cronbach’s *α* = 0.76–0.79), which included a total of 6 items on a 5-point Likert scale (1 = “not at all true for me,” 5 = “very true of me,” Neff et al., 2021).

The decentering audio was based on “Strenghtening the inner observer: thoughts and feelings are transient and not facts” (Faßbinder et al., 2015, p. 2019) by Faßbinder et al. (2015) and adapted from Mindfulness-based cognitive therapy (see Segal et al., 2012). The self-compassion intervention (SG) included an audio-guided loving-kindness meditation emphasizing self-kindness and shared humanity and was based on the Mindful Self-Compassion Workshop program by Neff and Germer (2013) (see Schug, 2016, p. 97). As in the study by Caldera (2017), the control group (CG) listened to a neutral audio recording presented in a monotone voice. To ensure participants remained attentive, comprehension questions were included. This active control condition helped to ensure intervention integrity and to rule out the possibility that differences between groups are merely due to relaxation or the act of listening to audio.

In our intervention study, we followed the recommendations outlined by Crane (2019), who emphasized the importance of ensuring intervention integrity and replicability in the context of mindfulness-based interventions (Crane, 2019). In line with this, we applied the Template for Intervention Description and Replication (TIDieR) as a guiding framework (see Crane, 2019). This approach informed our decision to investigate two existing, less-studied and less-validated forms of guided meditation (for an overview see Supplementary Table S1).

#### 3.1.4 Data analysis plan

Means and standard deviations were calculated for all trait and state variables by randomized group and assessment time (see Table 5). First, a multivariate analysis of covariance (MANCOVA) was conducted as the primary test of intervention effects. Group (Decentering Meditation [DG], Self-Compassion Meditation [SG], Control [CG]) served as the between-subjects factor. Post-intervention state decentering and post-intervention state self-compassion were entered as dependent variables, while the respective pre-intervention scores were included as covariates. The multivariate model tested overall group differences on the post-intervention outcomes while controlling for baseline levels. Where the multivariate effect of group was significant, follow-up univariate ANCOVAs were conducted separately for each outcome variable, again controlling for the corresponding pre-intervention score.

**Table 5:** Means (M) and standard deviations (sd) of the measured variables before (pre) and after intervention (post) for both intervention groups (DG: decentering, SG: self-compassion) and the control group (CG) from the raw data.

Variables		DG	SG	CG
I Trait variables		M, sd	M, sd	M, sd
Self-esteem	pre	3.01 (0.43)	3.25 (0.40)	3.02 (0.46)
Self-compassion	pre	3.07 (0.63)	3.28 (0.65)	3.18 (0.80)
Decentering	pre	3.18 (0.33)	3.28 (0.31)	3.10 (0.37)
II State variable		M, sd	M, sd	M, sd
Self-compassion	pre	3.27 (0.73)	3.73 (0.60)	3.38 (0.76)
	post	3.64 (0.67)	3.78 (0.57)	3.39 (0.71)
Decentering	pre	3.40 (0.69)	3.62 (0.63)	3.45 (0.91)
	post	4.01 (0.54)	4.05 (0.48)	3.56 (0.79)

Reported means are based on raw descriptive statistics (unweighted arithmetic means).

**Figure 4:**
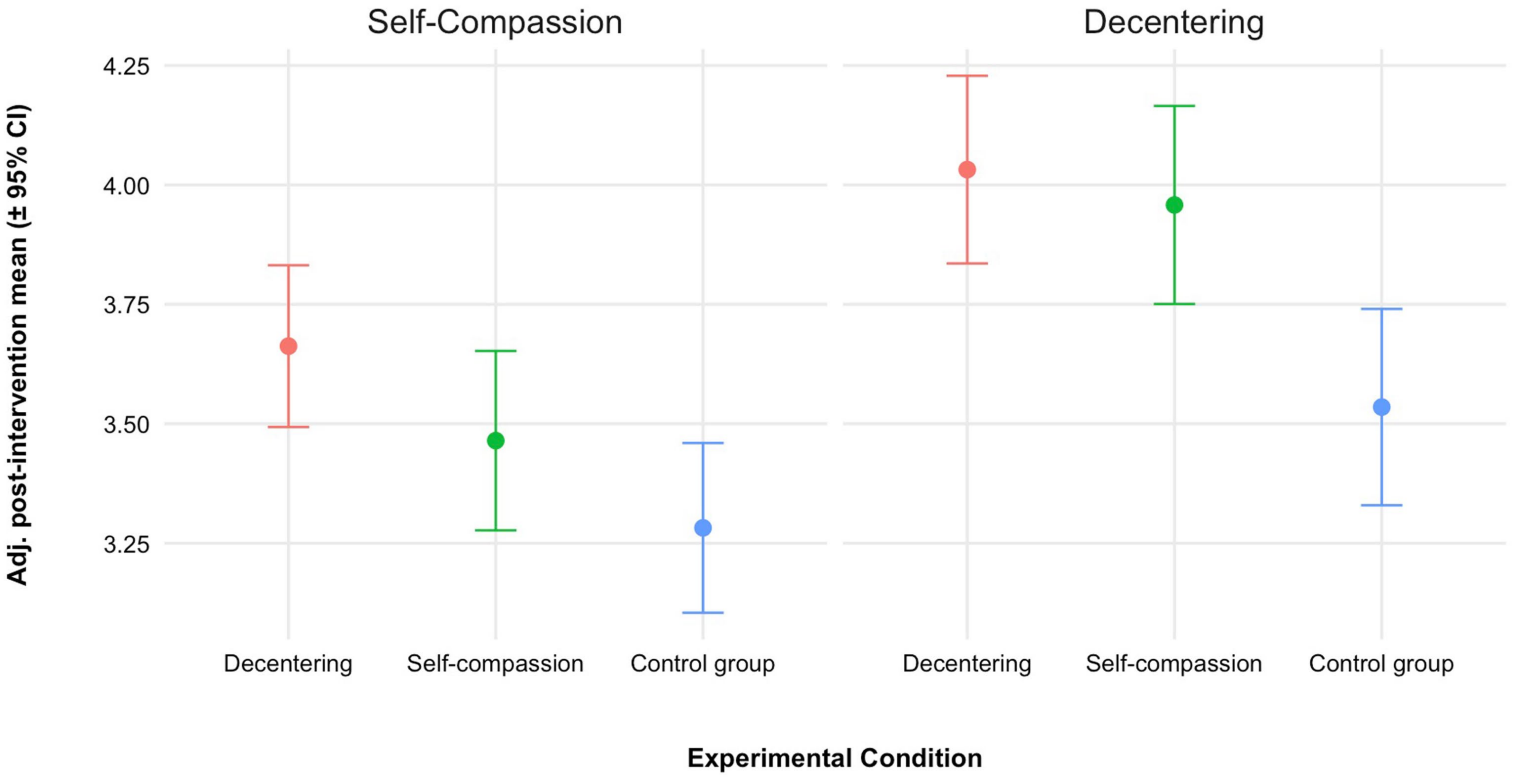
Adjusted post-intervention estimated marginal means (±95% CI) for each group (x-axis) and outcome, derived from ANCOVAs controlling baseline scores. Means represent group differences at post-intervention after accounting for initial levels. Self-Compassion and Decentering outcomes are shown in separate panels. Error bars represent 95% confidence intervals.

Following the univariate ANCOVAs, pairwise group comparisons were performed on the ANCOVA-adjusted estimated marginal means using Bonferroni correction; these results are visualized in Figure 4. For descriptive purposes only, trajectories of outcome scores over time based on model-based estimated marginal means from a linear interaction model including Group × Time × Variable can be found in the Supplementary material. As these estimates were not adjusted for baseline values, they represent absolute pre–post changes within each group (see Supplementary Figure S2).

To examine the predictive influence of trait variables on state outcomes, we conducted hierarchical linear regression analyses. For state self-compassion, the average of pre- and post-intervention scores was used, as these values were highly similar. In contrast, state decentering was analyzed separately at pre- and post-intervention, given observed differences across time points.

In the hierarchical regression models, trait self-esteem was entered in the first step, followed by trait decentering in the second step, and trait self-compassion in the final step. This approach allowed us to examine the incremental predictive value of each trait while accounting for variance explained by previously entered predictors. No interaction terms or time variables were included.

### 3.2 Results

#### 3.2.1 Multivariate analysis of covariance and group contrasts

The multivariate analysis of covariance (MANCOVA), controlling for baseline scores, revealed a significant multivariate effect of group, *Pillai’s trace* = 0.24, *F* (4,120) = 4.08, *p* = 0.003, *η^2^p* = 0.17, indicating that overall post-intervention outcomes in decentering and self-compassion differed between groups when adjusted for baseline levels. Baseline self-compassion and baseline decentering were strong covariates in the multivariate model, showing large partial effects on their respective post-intervention outcomes (self-compassion: *η^2^p* = 0.64; decentering: *η^2^p* = 0.43).

A univariate ANCOVA was conducted to examine group differences in post-intervention state self-compassion (Post-SC) in detail, with baseline self-compassion (Pre-SC) as a covariate. Results showed a significant main effect of group, *F* (2,61) = 4.79, *p* = 0.01, *η^2^p* = 0.16, indicating differences in adjusted post-intervention self-compassion across conditions and independent of initial levels of self-compassion. Baseline self-compassion was a strong predictor of post-intervention scores, *F* (1,61) = 100.99, *p* < 0.001, *η^2^p* = 0.62. Planned Bonferroni-adjusted pairwise comparisons revealed that the Decentering group reported significantly higher adjusted post-intervention self-compassion than the Control group (emmean = 3.66 vs. 3.28, *p* = 0.008). The Self-Compassion group (emmean = 3.46) scored descriptively higher than the Control (emmean = 3.28) and slightly lower than the Decentering group (emmean = 3.66), but neither difference reached statistical significance (*p* = 0.47 and *p* = 0.37, respectively). Both interventions were thus associated with higher self-compassion relative to the control condition, but only the Decentering group’s effect showed significance.

A second univariate ANCOVA was conducted to examine group differences in post-intervention state decentering (Post-MS), controlling for baseline state decentering (Pre-MS). The analysis revealed a significant effect of group, *F* (2,61) = 6.97, *p* < 0.01, *η^2^p* = 0.20, indicating differences in adjusted post-intervention decentering across conditions and independent of initial levels of decentering. Baseline state decentering was a strong covariate, *F* (1,61) = 48.44, *p* < 0.001, *η^2^p* = 0.44. Bonferroni-adjusted pairwise comparisons of estimated marginal means revealed that the Decentering group exhibited significantly higher adjusted post-intervention decentering than the Control group (emmean = 4.03, SE = 0.098 vs. 3.53, SE = 0.103; *p* = 0.003). The Self-Compassion group (emmean = 3.96, SE = 0.104) also showed significantly higher decentering levels than the Control group (emmean = 3.53, *p* = 0.016). However, no significant difference was observed between the Decentering and Self-Compassion groups (*p* = 1.00) after correction for multiple comparisons.

Table 5 shows the means and standard deviations of the measured variables before (pre) and after intervention (post) for both intervention groups and the control group (CG) from the raw data.

#### 3.2.2 Hierarchical regression analysis

Hierarchical multiple regression analyses were conducted to examine the predictive effects of self-esteem, decentering, and trait self-compassion on average state self-compassion. Model 1, including self-esteem, was significant (*F* (1, 63) = 42.46, *p* < 0.001, *R*^2^ = 0.40), with self-esteem emerging as a significant predictor (*b* = 0.94, *p* < 0.001). Model 2 significantly improved model fit compared to Model 1 (Δ*F* (1, 62) = 9.68, *p* = 0.003), explaining an additional 7% of variance (*R*^2^ = 0.47). In this model, both self-esteem (*b* = 0.65, *p* < 0.001) and decentering (*b* = 0.62, *p* = 0.006) were significant predictors. Model 3 significantly improved model fit compared to Model 2 (Δ*F* (1, 61) = 12.93, *p* < 0.001), increasing explained variance to *R*^2^ = 0.56. In the final model, trait self-compassion emerged as the only significant predictor (*b* = 0.51, *p* < 0.001), while self-esteem (*b* = 0.23, *p* = 0.253) and decentering (*b* = 0.24, *p* = 0.295) were no longer significant.

For pre-intervention state decentering, hierarchical regression analyses showed a different pattern. Self-esteem alone did not significantly predict pre-state decentering (Model 1: *F* (1, 64) = 2.33, *p* = 0.13, R^2^ = 0.035, b = 0.32, *p* = 0.13). Adding decentering in the second step significantly improved the model (Model 2: ΔR^2^ = 0.11, *F* (1, 63) = 7.96, *p* = 0.006), with decentering emerging as a significant predictor (b = 0.90, *p* = 0.006), whereas self-esteem remained non-significant (b = −0.10, *p* = 0.68). The addition of trait self-compassion in the third step did not further increase explained variance (Model 3: ΔR^2^ = 0.01, *F* (1, 62) = 0.70, *p* = 0.40). Thus, in the final model, trait decentering remained the only significant predictor of pre-intervention state decentering (b = 1.05, *p* = 0.006), while self-esteem (b = 0.06, *p* = 0.85) and self-compassion (b = −0.19, *p* = 0.40) were not significant. For post-intervention state decentering, hierarchical regression analyses revealed that none of the trait variables significantly predicted state decentering. Self-esteem alone showed a small, significant effect in the first model (Model 1: *F* (1, 63) = 4.43, *p* = 0.03, R^2^ = 0.066, b = 0.38, *p* = 0.03), but this effect did not remain when decentering and self-compassion were added. Adding decentering in Model 2 did not significantly increase explained variance (ΔR^2^ = 0.009, *F* (1, 62) = 0.62, *p* = 0.43), and neither self-esteem (b = 0.28, *p* = 0.22) nor decentering (b = 0.23, *p* = 0.43) were significant predictors. Finally, including trait self-compassion in Model 3 also failed to improve the model (ΔR^2^ = 0.001, *F* (1, 61) = 0.09, *p* = 0.76), with all predictors remaining non-significant (self-esteem: b = 0.33, *p* = 0.25; decentering: b = 0.28, *p* = 0.40; self-compassion: b = −0.06, *p* = 0.76).

### 3.3 Discussion study 2

Our second study investigated whether the prior investigated trainable variables decentering and self-compassion could be cultivated through two brief guided meditation interventions. It is known that even very short meditations, for instance 10 min over a period of 10 days, can lead to positive psychological changes and enhancements in mindfulness (Flett et al., 2019). For this reason, it is important to isolate easily implementable meditation techniques which can help people to enhance their decentering and self-compassion levels. Therefore, we adapted two guided meditations on Mindfulness-based Cognitive Therapy (see Faßbinder et al., 2015, p. 219) and Mindful Self-Compassion (see Schug, 2016, p. 97) from established psychotherapeutic resources.

The results indicate that both interventions were effective in enhancing these processes relative to a control condition, supporting the feasibility of brief, digitally delivered meditation formats. However, the effects differed across constructs. For state self-compassion, only the Decentering intervention led to a statistically significant increase compared to the control group, while the Self-Compassion intervention showed descriptively higher scores relative to the control group without reaching significance. In contrast, state decentering increased significantly in both interventions compared to the control condition, with no difference between active groups. This pattern suggests a substantial overlap between decentering and self-compassion processes.

Different regression analyses further clarified the relationship between dispositional and state-level processes. Hierarchical regression revealed that trait self-compassion was the strongest predictor of average state self-compassion, with self-esteem and decentering no longer contributing significantly once trait self-compassion was included. This finding underscores the close conceptual linkage between trait and state self-compassion. In contrast, trait decentering significantly predicted pre-intervention state decentering, whereas self-esteem and trait self-compassion did not. Following the intervention, none of the trait variables significantly predicted post-intervention state decentering, indicating that the brief meditation exercise reduced individual differences in state decentering and led to more homogeneous levels across participants. This pattern highlights the malleability of decentering as a psychological skill, in contrast to self-compassion, which appears more closely tied to dispositional levels.

Overall, these findings suggest that decentering is particularly trainable and may also contribute to increases in self-compassion. Specifically, the Decentering intervention enhanced both decentering and self-compassion significantly, whereas the Self-Compassion intervention significantly increased only decentering. This pattern indicates that decentering may act as a foundational mechanism that fosters broader adaptive processes. Further discussion of these results considering methodological limitations can be found in the General Discussion.

## 4 General discussion

In summary, both studies demonstrate that decentering as well as self-compassion play an important role in self-esteem regulation. While Study 1 emphasized the foundational associations between self-esteem regulation and decentering as well as self-compassion, Study 2 highlighted preliminary immediate effects of two single-session interventions cultivating these variables.

In line with Study 1 and Hypotheses 1 and 2, which proposed that decentering and self-compassion mediate the relationship between self-esteem and self-esteem regulation strategies and that meditation experience may moderate these effects, the results provided partial support for these assumptions.

First, initial multiple regression analyses showed that decentering was significantly associated with greater self-affirmation, whereas self-compassion was significantly associated with lower levels of self-protection strategies, highlighting the distinct roles of these two constructs.

Second, the path analysis following PROCESS logic revealed that self-esteem was positively associated with both decentering and self-compassion (a-paths), while at the b-path level, self-compassion was significantly linked to lower self-protection strategies. In the context of mediation effects, self-compassion significantly mediated the relationship between self-esteem and self-protection, while all other expected indirect effects in the path model, namely the effects of self-compassion on self-affirmation and of decentering on both self-affirmation and self-protection, did not reach significance.

Third, moderated mediation analyses examining whether meditation experience moderated the indirect effects observed in the path analysis revealed no significant interactions for any of the indirect paths, indicating that meditation experience did not alter the strength of these effects within the moderated mediation models. Nevertheless, inspection of conditional indirect effects revealed significant mediation effects of decentering in the association between self-esteem and self-affirmation, as well as of self-compassion in the associations between self-esteem and both self-protection and self-affirmation. This indicated that some effects that were only marginal in the aggregated path model reached significance under moderation, or rather in the context of the moderated mediation framework (see the “Limitations” section for further discussion).

Lastly, group comparisons using analyses of covariance indicated that observed differences between meditators and non-meditators in the study variables were significant for self-esteem, decentering, and self-compassion, whereas effects for self-affirmation and self-protection were attenuated and not significant. In this context, age showed consistent associations across outcomes, such that higher age was associated with higher self-esteem, decentering, self-compassion, and self-affirmation, and with lower self-protection. This suggests that age-related developmental, cognitive, or experiential factors may contribute independently to self-related processes and should therefore be considered when interpreting group differences in contemplative research. With this in mind, longitudinal and experimental designs are needed to disentangle pre-existing group differences from potential effects of meditation practice. Such designs would allow for stronger causal inferences regarding whether changes in decentering, self-compassion, and related constructs can be attributed to meditation training itself, rather than to self-selection effects or demographic confounding.

Building on the correlational findings of Study 1, Study 2 extended this work by examining whether decentering and self-compassion could be cultivated through brief guided meditation interventions, with results providing partial support for the study hypotheses.

Controlling for baseline levels, both interventions led to significant increases in state decentering relative to the control condition, while only the decentering intervention produced a significant increase in state self-compassion compared to the control group.

After this, hierarchical multiple regression analyses further clarified the associations between dispositional traits and state outcomes. For state self-compassion, self-esteem showed an initial effect, but only trait self-compassion remained significant in the final model. For state decentering, trait decentering predicted pre-intervention states, with no significant predictors post-intervention. These findings highlight the short-term malleability of decentering relative to self-compassion at the state level. Brief guided practices appear capable of reducing individual differences in decentering, producing immediate positive shifts, whereas trait influences on self-compassion remain to be explored in a more nuanced way. Future research should examine further inter-individual moderators (e.g., baseline mindfulness, self-criticism, affective stability) to clarify for whom and under which conditions brief meditation is most effective.

From a contemplative science perspective, findings from both studies lend empirical support to long-standing claims that meditation fosters both mental clarity and emotional equanimity (see Lomas et al., 2015; Eberth et al., 2019). They also raise intriguing questions for future research. For example, it would be valuable to investigate whether different forms of meditation (e.g., focused attention, open monitoring, loving-kindness) and belonging to specific groups (e.g., monastics vs. lay practitioners) differentially enhance decentering versus self-compassion and whether these, in turn, relate to different patterns of self-esteem regulation. In addition, momentary assessment methods, such as ecological momentary assessment (EMA) and other experimental paradigms, could be used to provide more nuanced insights into how individuals regulate self-esteem in real-life situations (see Naragon-Gainey et al., 2023). Moreover, the integration of these constructs into broader models of identity development and self-concept (e.g., individualism vs. collectivism, as in Hofstede) could additionally enrich our understanding of how metacognitive and compassionate capacities support psychological well-being.

Overall, the results add to the growing evidence supporting the use of brief, digitally delivered meditation interventions in everyday mental health practices, and highlight the important role of decentering and self-compassion in self-regulation. They also carry meaningful clinical implications. In particular, therapeutic approaches aimed at individuals with low self-esteem or heightened self-critical tendencies, such as Compassion-Focused Therapy (CFT) or Mindfulness-Based Cognitive Therapy (MBCT), could benefit from explicitly targeting decentering and self-compassion as regulatory skills. Furthermore, such training might not only be beneficial for clinical populations but could also serve a preventive and resilience strengthening role in non-clinical individuals, like students or young adults facing chronic stress, identity transitions, or performance pressure. With this in mind, it may be valuable to investigate whether interventions that combine decentering and self-compassion techniques yield synergistic effects, as well as how such approaches perform across diverse populations and delivery formats. This suggestion is supported by theoretical considerations but also correlational analyses (see the results section), indicating a close association between the two constructs (see Neff, 2023). This conceptual overlap, which was also seen by the VIF analyses, should therefore be considered when examining their effects in future research.

Lastly, it is important to note that the factor structure of the analyzed constructs warrants additional consideration when examining them in follow-up studies. Decentering, for instance, can be differentiated into an accepting self-perspective and a distanced perspective (Gecht et al., 2014; Rader et al., 2023). Investigating how these subfactors independently contribute to self-esteem regulation in future studies may provide further insight into the mechanisms underlying such combined interventions.

## 5 Limitations

Despite the promising findings in both of our studies, several limitations should be considered. First, the complexity of the statistical models in combination with the relatively small sample size in Study 1 may have reduced statistical power, particularly for detecting smaller indirect effects in the mediation framework and thus may have contributed to potential model overfitting. This issue is further reflected in the shared variance between decentering and self-compassion, as indicated by correlation and VIF analyses, which may have influenced the estimation of unique indirect effects in the parallel-mediator model. Together, these factors help explain why the moderated mediation and single-mediator robustness analyses were directionally consistent with the parallel model, yet differed in statistical detectability. Rather than indicating contradictory findings, this pattern likely reflects differences in model specification and statistical sensitivity, driven by a combination of limited statistical power, shared variance among mediators, and overall model complexity. Ultimately, the full parallel mediation model estimated multiple indirect effects simultaneously, while the moderated mediation analyses examined conditional processes in a more segmented framework, which may have increased sensitivity to detect context-dependent effects that are less apparent in the aggregate model.

Another key limitation to be considered is the reliance on self-report measures, as such measures are inherently susceptible to social desirability bias and limitations in introspective accuracy, potentially introducing common method bias. Future research should therefore address this limitation by employing multi-method designs and incorporating procedural and statistical approaches, such as marker-variable techniques, to reduce shared method variance. Future research, particularly intervention-based designs, should incorporate objective or physiological indicators alongside self-report measures to provide a more robust assessment of meditation-related processes. This is particularly important given that self-worth threats and subsequent self-regulation involve autonomic nervous system responses, which can be captured through heart rate variability (HRV) and galvanic skin response (GSR). HRV, reflecting the variation between successive heartbeats, is associated with physiological and psychological regulation, with higher HRV indicating greater regulatory capacity (Quirin et al., 2024). Previous studies have shown that mindfulness and related interventions can influence HRV (Christodoulou et al., 2020; Steffen et al., 2021). GSR on the other hand, serves as an objective measure of stress reactivity and relaxation levels (e.g., Bakker et al., 2011; Kurniawan et al., 2013), with higher mindfulness levels being linked to greater decrements in psychophysiological activation measured via GSR after stressful tasks (Dorjee, 2020; Scavone et al., 2020). Both HRV and GSR could therefore be used to validate the effectiveness of meditation interventions (see Arredondo et al., 2017) and to quantify differences in relaxation during such interventions.

In addition, the analyses of covariance in Study 1 constitute an additional limitation, as their results should be interpreted with caution due to their exploratory nature and sensitivity to assumption violations observed in preliminary analyses (non-linearity and variance heterogeneity for self-protection). Replication in larger, adequately powered samples is therefore needed to further establish robustness and generalizability.

Finally, concerning Study 2, it should be noted that participants were distributed across three experimental conditions, resulting in a limited effective sample size per group. Together with the overall relatively small sample size, this resulted in limited statistical power. Consequently, Study 2 may have lacked sufficient power to reliably detect small-to-moderate effects, increasing the risk of Type II errors and unstable parameter estimates. Therefore, the conclusions regarding the results of Study 2 remain tentative, similar to Study 1, as they have not yet been replicated in larger, adequately powered samples.

## 6 Conclusion

The present studies provide initial insights into the role of two key trainable psychological constructs: decentering and self-compassion in self-esteem regulation.

Study 1 demonstrates that both constructs are associated with the adoption of self-protective versus self-affirming self-esteem strategies. Although the moderation analyses did not reach statistical significance, analyses of covariance and mean differences indicate that individuals with meditation experience report higher levels of self-esteem, decentering and self-compassion compared to non-meditators. Furthermore, regression analyses suggest that decentering and self-compassion are meaningfully related to the use of these self-esteem strategies and that self-compassion is especially influential when analyzing self-protection. Taken together, these findings indicate that decentering and self-compassion play an important role in self-esteem regulation and that meditation experience can be associated with more adaptive patterns of self-esteem regulation, even if its moderating role requires further empirical clarification.

Study 2 extends these findings by showing that both decentering and self-compassion can be effectively enhanced through a single brief guided meditation session, resulting in measurable post-intervention increases compared to a control condition. Notably, the interventions also appeared to reduce trait-related variability in state decentering, indicating that brief meditation practices may temporarily attenuate individual differences and elevate participants to more homogeneous state levels. These results underscore the potential of short, low-threshold meditation formats as cost-effective and easily implementable tools for strengthening psychological processes relevant to self-regulation.

Taken together, the findings of both studies highlight decentering and self-compassion as important yet still underexplored mechanisms in self-esteem regulation. Future research should build on these initial results by employing experimental and longitudinal designs to assess the durability of intervention effects, clarify underlying mechanisms, and enhance the generalizability of the findings across clinical and non-clinical populations. Our findings emphasize and underline more effort to validate known short, guided meditation texts and create new ones from existing workbooks and manuals.

## Data Availability

Data supporting the conclusions of this article are available from the corresponding authors upon reasonable request and without undue reservation.

## Footnotes

1
https://qualtrics.com

2
https://r-project.org/
