# Supplementary material for: The role of decentering and self-compassion in self-esteem regulation: how meditation and metacognition shape the use of self-esteem regulation strategies
Source: Front Psychol. 2026 Jul 10;17:1791258. doi: 10.3389/fpsyg.2026.1791258 (PMC13408966; doi:10.3389/fpsyg.2026.1791258)
Supplement: Supplementary file 1 [file Supplementary_file_1.DOCX]

**Supplemental Material**

**Supplementary figure 1**

As indicated in the paper, a variable in addition to decentering and self-compassion, namely other-compassion, measured via the compassion for others scale (COS-7, Schlosser et al., 2023) was originally planned for inclusion and calculated. The exclusion of the variable due to considerations of poor model fit and to simplify the presentation was reached after comparing models with and without the COS. The comparison shows that the exclusion does not affect the main results or the mediation effects found. The model including other-compassion and the model excluding other-compassion are similar in their effect sizes and significances concerning the analyzed paths and the mediation effects (see Figure S1). The only significant mediation effect was for the effect of self-compassion on self-esteem and self-protection.

From a theoretical perspective, the non-significant results between other-compassion and self-esteem, despite expectations of a positive association, could be explained by the characteristics of the COS-7, which primarily measures selfless compassion and excludes self-enhancing or public prosocial components. The role of other-compassion may therefore be more complex than the initial model suggested. Studies have highlighted that other-compassion can serve self-enhancement purposes, functioning to elevate one’s self-image rather than reflecting purely altruistic motivation (see Surana & Lomas, 2014; Schlosser et al., 2023; Butler & Tomazos, 2011; Seo & Scammon, 2014; Sprecher & Fehr, 2006). If such other-compassion levels were to be captured, the COS-scale might not have been a suitable scale altogether. According to the authors, the COS-7 shows particularly strong correlations with *empathic concern*, *emotional prosocial behavior*, and *dire prosocial behavior* (Schlosser et al., 2023). The scale therefore captures not only the extent to which individuals are willing to help others in crisis or emotionally charged situations but, more importantly, whether they exhibit a general disposition toward compassion toward others, independent of public prosocial behavior (Schlosser et al., 2023). This may in part explain the non-significant associations with self-esteem observed in the present study. Future research could thus employ the COS-7 alongside other measures of other-compassion, particularly those that theoretically link other-compassion to self-esteem and self-enhancement (see Seo & Scammon, 2014), to ensure that different forms of other-compassion are encapsulated. The significant b3 path (other-compassion and self-affirmation) warrants future research.


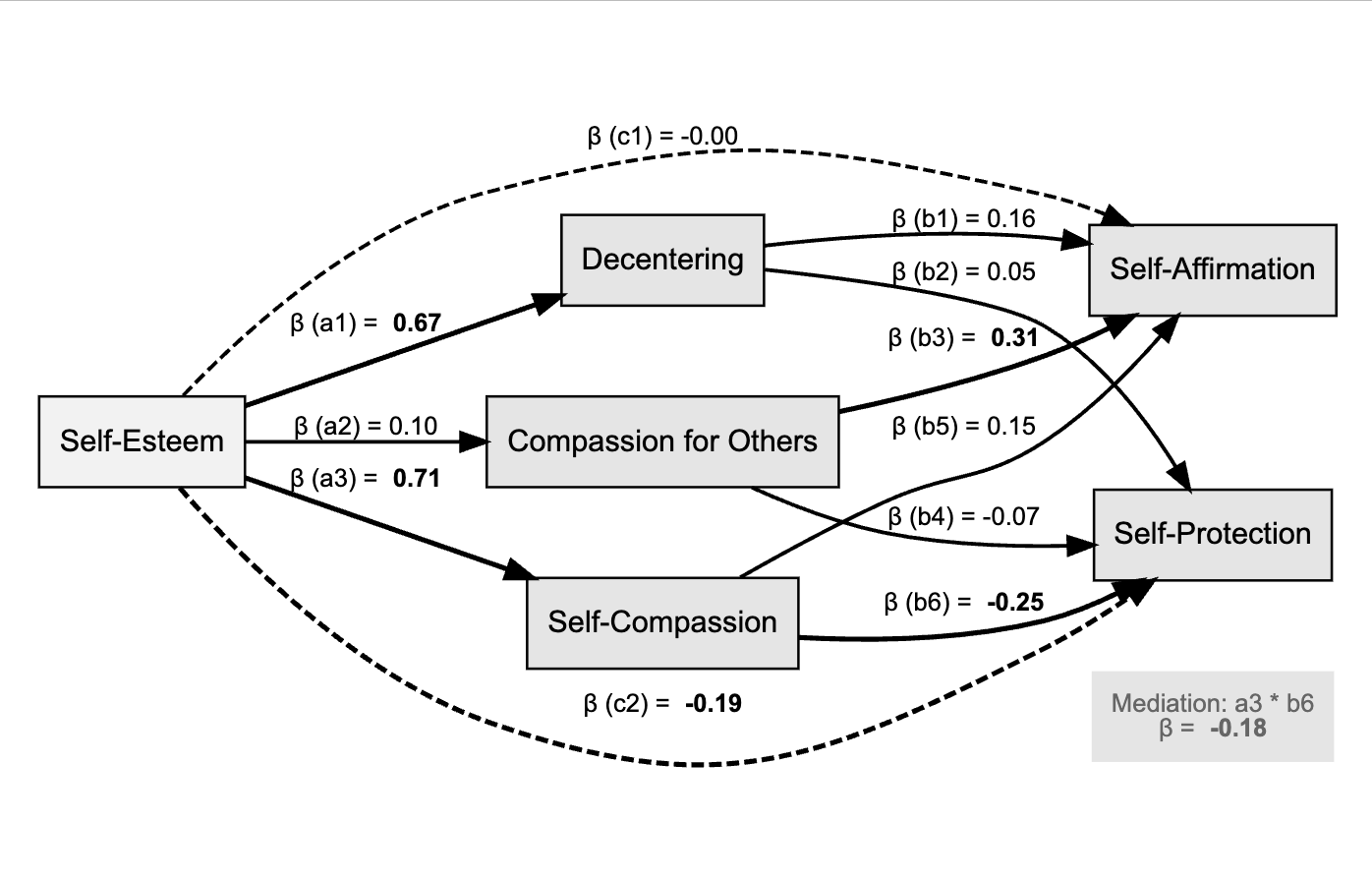
*Figure S1*. The path analysis, including other-compassion, shows that effects in the simplified model (without other-compassion) remain similar in effect sizes. Significant paths are indicated by bold lines.

**Supplementary figure 2**


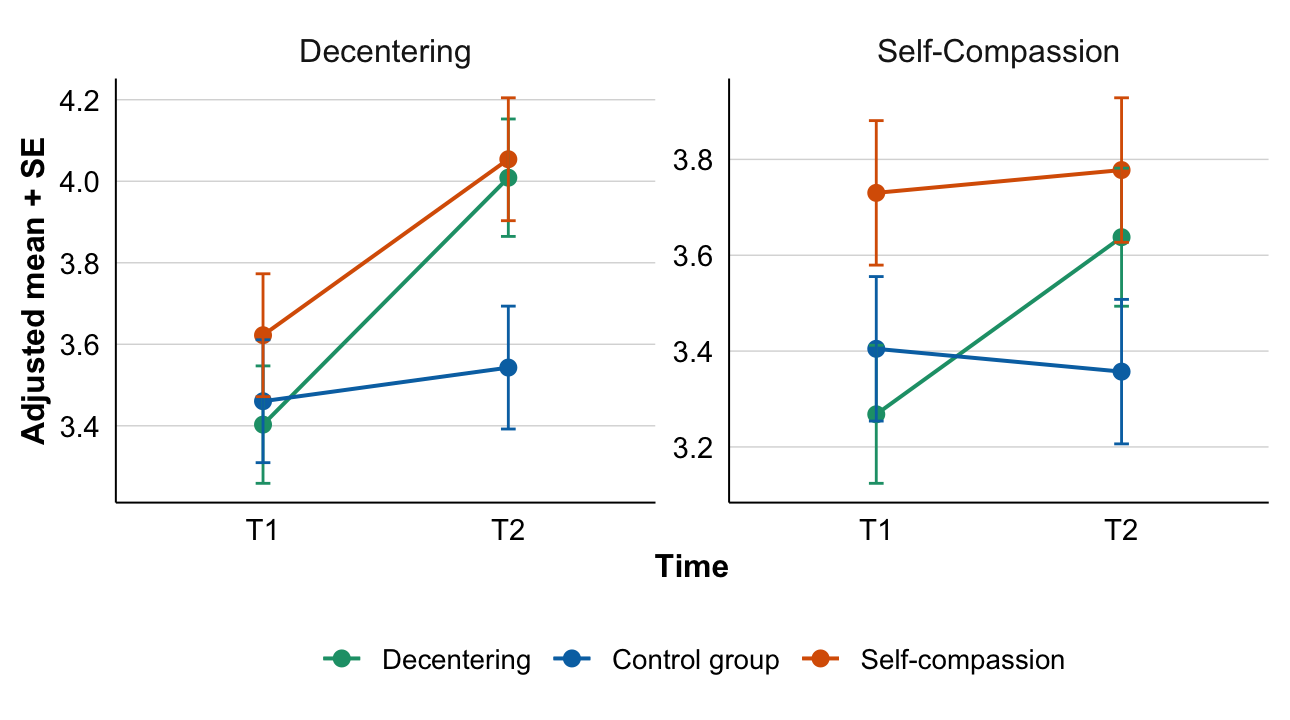


*Figure S2***.** Mean and standard error (SE) in state Decentering (on the left) and state Self-Compassion (on the right) among the Self-Compassion (orange), Decentering (green), and Control group (blue) before (T1) and after (T2) intervention. The Figure displays estimated marginal means (± SE) on the original 1–5 Likert scale.

**Supplementary figure 3**

**Table S1.** *Template for Intervention Description and Replication (TIDieR) according to Crane (2019).*

| **TIDieR Item** | **Meditation** |
| --- | --- |
|  | **Guided Meditation Interventions** |
| **Why (Rationale)** | to investigate if two lesser-known guided meditations (originally in text form) lead to short-term enhancements in decentering and self-compassion when applied via audio recordings. |
| **What (Materials and Procedures)** | An audio recording of a guided meditation based on a pre-existing written script under the name of “Den inneren Beobachter stärken: Gedanken und Gefühle sind vergänglich und keine Tatsachen!” (Faßbinder et al., 2015, p. 219) for decentering and “Metta-Meditation” or “Liebevolle Güte-Meditation” (see Schug, 2016. p. 97, based on Neff & Germer, 2013) for self-compassion. |
| **Whom Provided** | The instructor was a student of Clinical Psychology with a background in personal mindfulness practice under supervision of the Institute of Medical Psychology and Medical Sociology of the University Hospital RWTH Aachen. |
| **How (Delivery Mode)** | The guided meditations were pre-recorded and delivered via PC in lab |
| **Where** | Standardized in lab-sessions |
| **When and How Much** | one session for decentering and self-compassion respectively (each 17:29 minutes) |
| **Tailoring** | Participants in the decentering group received the decentering tailored meditation, while people allocated to the self-compassion group received the metta-meditation. |
| **Modifications** | No changes were made during the study. |
| **How Well (Planned & Actual Fidelity)** | Fidelity was accounted for through the control group (neutral audio, 15 minutes) and a comprehension test. The experiment instructor and recorder of all audio files were an experienced meditator and well versed in mindfulness. The audios were recorded professionally in a studio and are accessible upon request. |

**References**

Butler, R. und Tomazos, K. (2011) „Volunteer tourism: altruism, empathy or self enhancement?“, *New Problems in Tourism*, 1(4).

Faßbinder, E., Klein, J.P., Sipos, V. and Schweiger, U. (2015) *Therapie-Tools Depression: Mit E-Book inside und Arbeitsmaterial*. Weinheim: Beltz.

Schlosser, M. *u. a.* (2023) „The psychometric properties of the compassionate love scale and the validation of the English and German 7-item compassion for others scale (COS-7)“, *Current Psychology*, 42(1), pp. 579–591. https://doi.org/10.1007/s12144-020-01344-5.

Schug, S. (2016) *Therapie-Tools Achtsamkeit: Materialien für Gruppen- und Einzelsetting. Mit E-Book inside und Arbeitsmaterial*. Weinheim: Beltz.

Sprecher, S. und Fehr, B. (ohne Datum) „ENHANCEMENT OF MOOD AND SELF-ESTEEM AS A RESULT OF GIVING AND RECEIVING COMPASSIONATE LOVE“, 11(16).

Surana, P. und Lomas, T. (2014) „The power of charity: Does giving away money improve the wellbeing of the donor?“, *Indian Journal of Positive Psychology*, 5, pp. 223–230.

Yong Seo, J. und L. Scammon, D. (2014) „Does feeling holier than others predict good deeds? Self-construal, self-enhancement and helping behavior“, *Journal of Consumer Marketing*, 31(6–7), pp. 441–451. https://doi.org/10.1108/JCM-06-2014-1029.
